# Metabolic syndrome after liver transplant in patients at the specialized Center San Vicente Fundación, Rionegro, Antioquia, Colombia, 2013-2017

**DOI:** 10.17843/rpmesp.2022.394.11992

**Published:** 2022-12-22

**Authors:** Jorge Emilio Salazar-Flórez, Eliana María Arango-Flórez, Melissa Morales-Toro, Ana del Mar Rojas-Copete, Daniela Alejandra Leal-Castillo, Luis Guillermo Toro-Rendón

**Affiliations:** 1 School of Health Sciences, Medical Program, Fundación Universitaria San Martín, Sabaneta, Colombia. Universidad San Martín School of Health Sciences Medical Program Fundación Universitaria San Martín Sabaneta Colombia; 2 Epidemiology Group, Universidad CES, Medellín, Colombia. Universidad CES Epidemiology Group Universidad CES Medellín Colombia; 3 San Vicente Fundación Specialized Centers, Rionegro, Colombia. San Vicente Fundación Specialized Centers Rionegro Colombia

**Keywords:** Metabolic Syndrome, Liver Transplant, Colombia

## Abstract

The medical records of all liver transplant patients attended at the Centro Especializado San Vicente Fundación between January 2013 and June 2017 were reviewed in order to determine the frequency of post-transplant metabolic syndrome (MS). We collected sociodemographic data, pathological history, toxicological history, complications, and ATP III criteria in a validated instrument. The statistical analysis was carried out with OpenEpi 3.01; p<0.05 was considered as statistically significant. Of the 102 reviewed medical records, 73 met the inclusion criteria (no MS diagnosis prior to transplant and complete information for the instru­ment) and were analyzed. Most patients were male (59%), older adults (64%) and married (62%). The frequency of MS after liver transplant was 66%. The association between MS and history of hypertension and diabetes was significant. We confirmed that MS is a frequent complication in liver transplant recipients and that history of hypertension and diabetes are the most frequent associated factors.

## INTRODUCTION

After renal transplantation, liver transplantation is the most frequent in the world; according to the Global Observatory on Donation and Transplantation, 139,024 organ transplants were performed in 2017, of which 65% were renal transplants and 23% were liver transplants ^1^. Specifically in Colombia, for the year 2017, a total of 1342 patients received solid organ transplantation, of which 21% corresponded to liver transplantation ^2^. Regional # 2 of the National Donation and Transplantation Network that includes the authorized institutions of the departments of Antioquia, San Andrés and Providencia, Chocó, Córdoba, and Caldas, was the second in terms of number of donors and transplants performed, with 334 transplants, of which 19% were liver transplants. In addition, the Centro Especializado San Vicente Fundación Rionegro performed the most transplants in 2017 within the five specialized centers with 122 transplants; 6% of these were liver transplants ^2^. 

The optimization of the surgical technique and immunosuppressive treatment has made it possible to achieve excellent survival rates after liver transplantation, reaching 90% after one year and 80% after five years; nevertheless, this high survival rate is accompanied by an increase in medical complications such as the development of *de novo* neoplasms, recurrence of the underlying disease and metabolic and cardiovascular complications, which currently are the main causes of death unrelated to the graft ^3^. 

Metabolic syndrome (MS) is a chronic condition in which metabolic abnormalities coexist, such as central obesity, increased triglycerides, atherogenic dyslipidemia, hyperglycemia, and arterial hypertension, which constitutes risk factors for developing cerebrovascular events and type 2 diabetes mellitus ^4^ ^,^ ^5^. Several international organizations and scientific groups have defined the diagnostic criteria for MS, such as the National Cholesterol Education Program - Adult Treatment Panel III (NCEP- ATP III), the World Health Organization, the International Diabetes Federation and the European Study Group on Insulin Resistance (EGIR); being the criteria of the International Diabetes Federation and the Adult Treatment Panel III (ATP III) in its modified version 2015, the most widely used criteria for the diagnosis of MS ^4^. The ATP III defined the presence of three of the following five factors as determinants for the diagnosis of MS: a) abdominal obesity (>102 cm in men and >88 cm in women) measured by abdominal perimeter; b) hypertriglyceridemia >150 mg/dL, c) low HDL levels (men <40mg/dL and women HDL <50 mg/dL); d) blood pressure >130/85 mmHg; e) glycemia >100 mg/dL (5.6 mmol/L).

Previous studies report that the prevalence of MS ranges from 44% to 58% in liver transplant patients ^3^ ^,^ ^6^ ^,^ ^7^. Furthermore, it has been reported that MS increases 1.78 times the risk of developing cardiovascular disease and death, and this syndrome, together with immunosuppression, represents the main risk factor for the development of cardiovascular disease, which is associated in 19% to 42% of the cases to all deaths not associated with the graft ^3^, which increases the use of resources allocated to health care due to an increase in the number of hospitalizations during the first year after liver transplantation ^8^ ^,^ ^9^.

There are no clear data from Colombia on the prevalence of MS in liver transplant patients, which limits the available knowledge on the epidemiological behavior of this syndrome, besides, there are no studies carried out in patients from the San Vicente Fundación Hospital in Rionegro that could allow us to establish a relationship between the appearance of MS and risk factors associated with this population. This information is important, because it could help address the modifiable risk factors in order to avoid the appearance of the syndrome or to minimize the negative impact on the selected group of patients, thus reducing morbidity and mortality and improving the quality of life, both physically and psychologically, since this syndrome can affect the life style of patients. In addition, timely intervention reduces the demand for health resources.

This study aimed to determine the frequency of MS in liver transplant patients from the Centro Especializado San Vicente Fundación Rionegro between 2013 and 2017, to describe the sociodemographic characteristics of these patients, and to explore the association of post-transplant MS with possible risk factors in the studied population.

**KEY MESSAGES**

Motivation for the study: there is a lack of studies in Latin America on the frequency of metabolic syndrome in patients who receive liver transplants.

Main findings: two-thirds (66%) of patients who received liver transplantation between 2013 and 2017 at the Specialized Center San Vicente Fundación de Rionegro, Antioquia, Colombia, subsequently presented metabolic syndrome.

Implications: this study confirms that liver transplant recipients very frequently develop metabolic syndrome; however, the frequency found by this study (66%) was almost double that reported in other regions of the world, suggesting that patients from the Specialized Center San Vicente Fundación de Rionegro, Antioquia, Colombia, may present some additional condition.

## THE STUDY

### Type of study and population

Retrospective observational study conducted on liver transplant patients from the Centro Especializado San Vicente Fundación Rionegro between January 2013 and June 2017.

### Inclusion and exclusion criteria

We included the medical records of all patients who underwent liver transplantation between January 2013 and June 2017. Patients who were diagnosed with MS prior to transplantation and those who had medical records with insufficient information were excluded from the analysis.

### Collection of information

Authorization was requested from the Centro Especializado San Vicente Fundación Rionegro in order to use the information from the medical records of the liver transplant patients who were treated between January 2013 and June 2017. We reviewed the medical records and applied an instrument validated by experts, which consisted of a registration form where information was collected on sociodemographic variables, pathological history, toxicological history, complications, and established ATP III criteria. The expert validation consisted of the independent review by three internal medicine professionals, of each of the items of the instrument. As part of the validation, the concepts were reviewed and changes were applied to the instrument. With this information, we created an Excel database that was reviewed by three researchers.

### Variables

The sociodemographic variables included in the instrument were: gender (male/female), age (adult/elder), race (white/mestizo/black), schooling (primary school or below/upper-secondary education/technical or higher), marital status (single or common-law/married/separated or widowed), occupation (employed/unemployed/pensioned) and health insurance (contributory/subsidized/special). The variables included regarding pathological and toxicological history and complications were: period of transplantation (semester), indication for transplantation (diagnosis), immunosuppressive therapy (drugs), cause of death (diagnosis), arterial hypertension (yes/no), diabetes mellitus (yes/no), dyslipidemia (yes/no), cardiovascular disease (yes/no), alcohol consumption (yes/no), cigarette smoking (yes/no).

### Statistical analysis

Online Openepi software version 3.01, and Office Excel were used for data analysis. Univariate analysis was carried out by calculating absolute frequencies and relative frequencies. The Chi-square test or Fisher’s exact test, as appropriate for the calculation of expected values, were used for the bivariate analysis. Statistical significance was considered as a p-value of less than 0.05.

### Ethical considerations

The project was evaluated and approved by the research committee of the Fundación Universitaria San Martín, act 010 of 2017. The informed consent and confidentiality agreement were signed with the Centro Especializado San Vicente Fundación de Rionegro.

## FINDINGS

**Figure 1:**
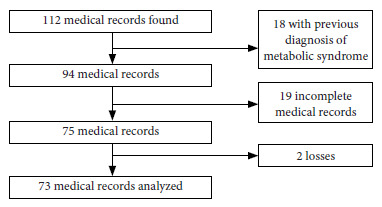
Diagram of medical record selection in order to calculate the frequency of metabolic syndrome in liver transplant recipients at the Hospital San Vicente Fundación, Rionegro, Antioquia, Colombia, 2013-2017.

A total of 112 medical records corresponding to all liver transplant recipients who attended the institution between January 2013 and June 2017 were reviewed. Of these, 39 were excluded from the analysis due to the following reasons (Figure 1): 18, because of previous MS diagnosis; 19, because they did not have enough information for the diagnosis of MS; and 2 because the patients were transferred to another hospital and could not be followed-up, so they were considered as losses.

A total of 73 medical records were included in the analysis; the frequency of MS was 65.8%. The sociodemographic variables of the patients are described in Table 1. Most were men (59%), elder (64%), mestizos (90%) and were married (62%). Most patients (37%) had upper-secondary education and 43% were employed.

**Table 1:** Sociodemographic characteristics of liver transplant patients at Hospital San Vicente Fundación, Rionegro, Antioquia, Colombia, 2013-2017.

Variable	n (%)
Sex	
Male	43 (58.9)
Female	30 (41.1)
Age	
Adult (18 to 59 years)	26 (35.6)
Elder (≥60 years)	47 (64.4)
Race	
White	4 (5.5)
Mestizo	66 (90.4)
Black	3 (4.1)
Schooling	
Primary school or below	24 (32.9)
Baccalaureate	27 (37.0)
Technical or higher	17 (23.3)
No data	5 (6.8)
Marital status	
Single or common-law	17 (23.3)
Married	45 (61.6)
Separated, divorced or widowed	11 (15.1)
Occupation	
Employed	31 (42.5)
Unemployed	26 (35.6)
Pensioner	14 (19.2)
No data	2 (2.7)
Health insurance	
Subsidized	10 (13.7)
Contributive	61 (83.6)
Special	2 (2.7)

Cryptogenic cirrhosis (19%) and hepatocellular carcinoma (14%) were the most frequent indications for liver transplantation (Table 2). Regarding pathologic history prior to transplantation, arterial hypertension was found in 32% of the patients, diabetes in 23%, dyslipidemia in 14% and cardiovascular disease in 6%; in addition, 36% of the patients consumed alcohol and 25% smoked cigarettes.

**Table 2:** Clinical characteristics of liver transplant patients at Hospital San Vicente Fundación, Rionegro, Antioquia, Colombia, 2013-2017.

Variable	n (%)
Transplant period	
2013	7 (9.6)
2014	15 (20.5)
2015	24 (32.9)
2016	20 (27.4)
2017-I	7 (9.6)
Indication for transplantation ^a^	
Fatty liver disease	17 (17.9)
Non-alcoholic steatohepatitis	10 (10.5)
Hepatitis C infection	3 (3.2)
Hepatitis B infection	3 (3.2)
Autoimmune hepatitis	5 (5.3)
Primary biliary cholangitis	13 (13.7)
Hepatocellular carcinoma	18 (18.9)
Cryptogenic cirrhosis	19 (20.0)
Other	7 (7.3)
Immunosuppressive therapy ^a^	
Prednisone	33 (18.8)
Tacrolimus	45 (25.6)
Ciclosporin	21 (11.9)
Mycophenolate	50 (28.4)
Azathioprine	9 (5.1)
Everolimus	18 (10.2)
Cause of death	
Hypovolemic shock	1 (16.6)
Septic shock	4 (66.7)
Renal insufficiency	1 (16.7)

a Categories are not exclusive

The year with the highest number of transplants was 2015, with 16%. The most commonly used drugs in immunosuppressive therapies were mycophenolate (50 patients), tacrolimus (45 patients) and prednisone (33 patients), but it should be considered that each patient had several drugs within their scheme so the association between immunosuppressive treatment and MS could not be evaluated. Six deaths were reported (8% mortality) during the follow-up period of this study; the most frequent cause of death was septic shock (67%) (Table 2).

Table 3 describes the bivariate analysis; after which we found only an association between the appearance of MS and the history of arterial hypertension (p=0.005) and diabetes (p=0.013).

**Table 3:** Factors associated with the occurrence of metabolic syndrome after liver transplantation in patients from the Hospital San Vicente Fundación, Rionegro, Antioquia, Colombia, 2013-2017.

Exposure factor (background)	Metabolic syndrome		p-value
	Yes	No	
Arterial hypertension			0.005 ^a^
Yes	20	3	
No	28	22	
Diabetes mellitus			0.013 ^a^
Yes	15	2	
No	33	23	
Dyslipidemia			0.260 ^b^
Yes	8	2	
No	40	23	
Cardiovascular disease			0.377 ^b^
Yes	3	1	
No	45	24	
Alcohol			0.163 ^a^
Yes	19	7	
No	29	18	
Cigarette			0.214 ^a^
Yes	13	5	
No	29	18

a Chi-square test

b Fisher’s exact test

## DISCUSSION

Two thirds of liver transplant patients from the Hospital de San Vicente Fundación de Rionegro, Antioquia, Colombia, between 2013 and 2017, developed MS after transplantation. The present study confirms, once again, that liver transplant patients very frequently develop MS; however, our results showed that the prevalence of MS (66%) was almost double that reported by Thoefner *et al*. in a systematic review and meta-analysis carried out in 2015 that included 16 papers and 3,539 liver transplant patients, in whom they found a prevalence of MS of 39% (range 16-64%) and an incidence of MS of 35% (range 21-49%) ^10^. This suggests that patients from the Centro Especializado San Vicente Fundación de Rionegro, Antioquia, Colombia, may present some additional condition, which should be studied, that makes them more likely to develop MS compared to liver transplant recipients from other regions of the world.

We found that history of arterial hypertension was a factor associated to the appearance of MS after liver transplantation, which coincides with that reported by Sprinzl *et al.* ^11^ on European patients and Couto *et al*. ^12^ on Hispanic and non-Hispanic patients, but contrasts with other studies that did not find such an association with patients from different regions of the world ^13^ ^-^ ^16^.

Previous history of diabetes was the other factor associated with the development of post-liver transplantation MS found by our study. This is consistent with previous reports from around the world ^11^ ^-^ ^19^ as well as with the meta-analysis by Thoefner *et al*. ^10^, which confirms the high impact of history of diabetes on the development of MS after liver transplantation (OR = 4.03; 95%CI = 2.81-5.80; I^2^ = 0%).

In this study it was not possible to evaluate the association between immunosuppressive therapy and the incidence of MS because the therapy includes the combination of several drugs and the patients underwent changes in both doses and drugs within treatment. However, the systematic review by Thoefner *et al*. ^10^ found that immunosuppressive drugs are not a risk factor for MS after liver transplantation.

There are two main limitations for this study. First, we could only include a limited number of medical records (73 of 112), because only those had complete information. The second limitation is that it was not possible to include obesity as a variable during the analysis because no information on the body mass index or abdominal perimeter of the patients was found in the medical records.

In conclusion, the prevalence of post-liver transplant MS in the Centro Especializado San Vicente Fundación de Rionegro, Antioquia, Colombia is high, even higher than the prevalence reported in other regions of the world. History of arterial hypertension and diabetes are the most relevant associated factors for the development of this syndrome.
