# Supplementary material for: Metabolic syndrome after liver transplant in patients at the specialized Center San Vicente Fundación, Rionegro, Antioquia, Colombia, 2013-2017
Source: Rev Peru Med Exp Salud Publica. 2022 Dec 22;39(4):474–9. doi: 10.17843/rpmesp.2022.394.11992 (PMC11397675; doi:10.17843/rpmesp.2022.394.11992)
Supplement: Translation

# Translation: Síndrome metabólico postrasplante hepático en pacientes del Centro Especializado San Vicente Fundación, Rionegro, Antioquia, Colombia, 2013-2017

**Authors:** Jorge Emilio Salazar-Flórez, Eliana María Arango-Flórez, Melissa Morales-Toro, Ana del Mar Rojas-Copete, Daniela Alejandra Leal-Castillo, Luis Guillermo Toro-Rendón

**Affiliations:** 1 Facultad de Ciencias de la Salud, Programa de Medicina, Fundación Universitaria San Martín, Sabaneta, Colombia.; 2 Grupo de Epidemiología, Universidad CES, Medellín, Colombia.; 3 Centros Especializados San Vicente Fundación, Rionegro, Colombia.

**Keywords:** Síndrome Metabólico, Trasplante Hepático, Colombia

## Abstract

Se revisaron las historias clínicas de todos los pacientes con trasplante hepático atendidos en el Centro Especializado San Vicente Fundación entre enero de 2013 y junio de 2017, para determinar la frecuencia de síndrome metabólico (SM) postrasplante. En instrumento validado se registraron datos sociodemográficos, antecedentes patológicos, antecedentes toxicológicos, complicaciones y criterios ATP III. Se utilizó OpenEpi 3,01 para análisis estadístico; se consideró significancia estadística con p<0,05. De 102 historias clínicas revisadas, se analizaron 73 que cumplieron criterios de inclusión (sin diagnóstico de SM pretrasplante y con información completa para el instrumento). La mayoría de los pacientes eran hombres (59%), adultos mayores (64%) y casados (62%). La frecuencia de SM postrasplante fue 66%; hubo asociación significativa entre SM y antecedentes de hipertensión arterial y diabetes. Se confirma que el SM es una complicación frecuente de los trasplantados hepáticos y los antecedentes de hipertensión y diabetes son los factores asociados más frecuentes.

## INTRODUCCIÓN

Después del trasplante renal, el trasplante hepático es el más frecuente del mundo; según el Observatorio Global sobre Donaciones y Trasplantes, en el año 2017 se realizaron 139 024 trasplantes de órganos, de los cuales 65% fueron trasplantes renales y 23% fueron trasplantes hepáticos ^1^. Específicamente en Colombia, para el año 2017, un total de 1 342 pacientes recibieron trasplante de órgano sólido, de los cuales 21% correspondieron a trasplante de hígado ^2^ ^) ^; la regional # 2 de la Red Nacional de Donación y Trasplantes que incluye las instituciones habilitadas de los departamentos de Antioquia, San Andrés y Providencia, Chocó, Córdoba y Caldas, fue la segunda en cuanto a número de donantes y trasplantes realizados, con una cifra de 334 trasplantes, de los cuales 19% fueron hepáticos. Además, dentro de los cinco centros especializados que más trasplantes realizaron en 2017 se encuentra el Centro Especializado San Vicente Fundación Rionegro, donde se hicieron 122 trasplantes y 6% de estos fueron de hígado ^2^.

La optimización de la técnica quirúrgica y del tratamiento inmunosupresor ha permitido alcanzar actualmente excelentes tasas de supervivencia postrasplante hepático, llegando a ser de 90% en un año y de 80% en cinco años; pero esa alta tasa de supervivencia se acompaña de aumento en las complicaciones médicas como desarrollo de neoplasias de novo, recurrencia de la enfermedad subyacente y complicaciones metabólicas y cardiovasculares, que hoy constituyen las principales causas de muerte no relacionadas con el injerto ^3^.

El síndrome metabólico (SM) es una entidad clínica crónica en la que coexisten anormalidades metabólicas, tales como obesidad de distribución central, elevación de los triglicéridos, dislipidemia aterogénica, hiperglucemia e hipertensión arterial, que constituyen factores de riesgo para desarrollar eventos cerebrovasculares y diabetes mellitus tipo 2 ^4^ ^,^ ^5^. Varias organizaciones o grupos científicos internacionales han definido los criterios diagnósticos de SM, entre los que se destacan el Programa Nacional de Educación en Colesterol - Adult Treatment Panel III (NCEP- ATP III), la Organización Mundial de la Salud, la Federación Internacional de Diabetes y el Grupo Europeo de Estudio de Resistencia a la Insulina (EGIR); siendo los criterios de la Federación Internacional de Diabetes y del Adult Treatment Panel III (ATP III) en su versión modificada 2015, los más utilizados globalmente para el diagnóstico de SM ^4^. El ATP III definió la presencia de tres de los siguientes cinco factores como determinantes para el diagnóstico de SM: a) obesidad abdominal (>102 cm en hombres y >88 cm en mujeres) medida por perímetro abdominal; b) hipertrigliceridemia >150 mg/dL, c) niveles bajos de HDL (hombres <40mg/dL y mujeres HDL <50 mg/dL); d) presión arterial >130/85 mmHg; e) glicemia >100 mg/dL (5,6 mmol/L).

En pacientes trasplantados hepáticos se han reportado prevalencias de SM que varían entre 44% y 58% ^3^ ^,^ ^6^ ^,^ ^7^. Además, se ha reportado que el SM representa 1,78 veces más riesgo de desarrollar enfermedades cardiovasculares y muerte y este síndrome junto con la inmunosupresión, representan el principal factor de riesgo para el desarrollo de enfermedad cardiovascular, la cual tiene una asociación del 19% al 42% con todas las muertes no asociadas al injerto ^3^, lo que incrementa el uso de los recursos asignados a la atención en salud por un aumento en el número de hospitalizaciones en el primer año después del trasplante hepático ^8^ ^,^ ^9^. 

En Colombia no hay datos claros sobre la prevalencia de SM en pacientes trasplantados hepáticos, lo cual genera una limitación cuando se pretende conocer el comportamiento epidemiológico de este síndrome, así como tampoco se cuenta con estudios realizados en los pacientes atendidos en el Hospital de San Vicente Fundación en Rionegro que nos permita establecer alguna relación entre la aparición del SM con factores de riesgo asociados a dicha población. El conocimiento de estos datos es importante debido a que con esta información se pueden intervenir factores de riesgo modificables que eviten la aparición del síndrome o que minimicen el impacto negativo en el grupo de pacientes seleccionados, permitiendo así disminuir la morbimortalidad y mejorar la calidad de vida, tanto física como psicológicamente, ya que la aparición de este síndrome puede afectar el desarrollo normal de la vida de las personas que lo padecen. Además, la intervención oportuna permite disminuir la demanda de recursos en salud. 

Este estudio tuvo como objetivos determinar la frecuencia de SM en pacientes con trasplante hepático atendidos en el Centro Especializado San Vicente Fundación Rionegro entre 2013 y 2017, describir las características sociodemográficas de estos pacientes y explorar la asociación de SM postrasplante con los posibles factores presentes en la población estudiada.

**MENSAJES CLAVE**

Motivación para realizar el estudio: en Latinoamérica hay escasez de estudios sobre la frecuencia con que los pacientes que reciben trasplantes de hígado luego desarrollan síndrome metabólico.

Principales hallazgos: dos tercios (66%) de los pacientes que recibieron trasplante hepático entre 2013 y 2017 en el Centro Especializado San Vicente Fundación de Rionegro, Antioquia, Colombia, presentaron posteriormente síndrome metabólico.

Implicancias: este trabajo confirma nuevamente que los trasplantados hepáticos desarrollan con mucha frecuencia síndrome metabólico; sin embargo, la frecuencia encontrada en este estudio (66%) fue casi el doble que la reportada en otras regiones del mundo, lo que sugiere que los pacientes atendidos en el Centro Especializado San Vicente Fundación de Rionegro, Antioquia, Colombia, pueden presentar alguna condición adicional.

## EL ESTUDIO

### Tipo de estudio y población

Estudio observacional retrospectivo realizado con pacientes trasplantados hepáticos que fueron atendidos en el Centro Especializado San Vicente Fundación Rionegro entre enero de 2013 y junio de 2017.

### Criterios de inclusión y exclusión

Se revisaron las historias clínicas de todos los pacientes que fueron sometidos a trasplante hepático entre enero de 2013 y junio de 2017. Se excluyeron del análisis los pacientes que tuvieron un diagnóstico de SM previo al trasplante y los que tenían historias clínicas con información insuficiente.

### Recolección de la información

Primero se solicitó autorización del Centro Especializado San Vicente Fundación Rionegro para hacer uso de la información consignada en las historias clínicas de los pacientes trasplantados hepáticos que fueron atendidos entre enero de 2013 y junio de 2017. Luego, se hizo la revisión de las historias clínicas y se diligenció un instrumento validado por expertos que correspondió a un formato de registro en la que se recabo información sobre variables sociodemográficas, antecedentes patológicos, antecedentes toxicológicos, complicaciones y criterios establecidos ATP III. La validación de expertos consistió en la revisión de cada uno de los ítems del instrumento por tres profesionales de Medicina Interna, por separado. Dentro de la validación se recogieron los conceptos emitidos y se introdujeron cambios en el instrumento. Con esta información se hizo una base de datos en Excel que fue revisada por tres investigadores. 

### Variables

Las variables sociodemográficas consignadas en el instrumento fueron: género (masculino/femenino), edad (adultez/vejez), raza (blanco/mestizo/negro), escolaridad (primaria o menos/bachillerato/técnica o superior), estado civil (soltero o unión libre/casado/separado o viudo), ocupación (empleado/desempleado/pensionado) y aseguramiento al régimen de salud (contributivo/subsidiado/especial). Las variables consignadas sobre antecedentes patológicos, toxicológicos y complicaciones fueron: período del trasplante (semestre), indicación para el trasplante (diagnóstico), terapia inmunosupresora (medicamentos), causa de muerte (diagnóstico), hipertensión arterial (si/no), diabetes mellitus (si/no), dislipidemia (si/no), enfermedad cardiovascular (si/no), consumo de alcohol (si/no), consumo de cigarrillo (si/no).

### Análisis estadístico

Para el análisis de datos se utilizó el software Openepi, versión 3,01, en línea y Excel de Office. Se realizó el análisis univariado por medio del cálculo de frecuencias absolutas y frecuencias relativas. El análisis bivariado se realizó con la prueba Chi cuadrado o la prueba exacta de Fisher, según correspondía por el cálculo de los valores esperados. Se consideró significancia estadística con valor p menor a 0,05.

### Consideraciones éticas

El proyecto fue evaluado y aprobado por el comité de investigaciones de la Fundación Universitaria San Martín, acta 010 de 2017. Se firmó consentimiento informado y acta de confidencialidad con el Centro Especializado San Vicente Fundación de Rionegro.

## HALLAZGOS

En total se revisaron 112 historias clínicas que correspondían a todos los trasplantados hepáticos que fueron atendidos entre enero de 2013 y junio de 2017 en la institución; de éstas se excluyeron del análisis 39, por las siguientes razones (figura 1): 18 por diagnóstico previo de SM, 19 porque no tenían información suficiente para hacer el diagnóstico de SM y 2 porque los pacientes fueron trasladados a otro hospital y no se pudo realizar el seguimiento, por lo que se consideraron pérdidas.

**Figura 1:** . Diagrama de selección de historias clínicas para calcular la frecuencia de síndrome metabólico en trasplantados hepáticos del Hospital San Vicente Fundación, Rionegro, Antioquia, Colombia, 2013-2017.

En total se incluyeron 73 historias clínicas en el análisis y se encontró una frecuencia de SM del 65,8%. Las variables sociodemográficas de los pacientes se describen en la tabla 1. La mayoría eran hombres (59%), adultos mayores (64%), mestizos (90%) y casados (62%). El nivel de escolaridad fue bachillerato o menos en 37% de los pacientes y 43% eran empleados.

**Tabla 1:** Características sociodemográficas de los pacientes trasplantados hepáticos del Hospital San Vicente Fundación, Rionegro, Antioquia, Colombia, 2013-2017.

Variable	n (%)
Sexo	
Masculino	43 (58,9)
Femenino	30 (41,1)
Edad	
Adultez (18 a 59 años)	26 (35,6)
Vejez (≥60 años)	47 (64,4)
Raza	
Blanco	4 (5,5)
Mestizo	66 (90,4)
Negro	3 (4,1)
Escolaridad	
Primaria o menos	24 (32,9)
Bachillerato	27 (37,0)
Técnica o superior	17 (23,3)
Sin dato	5 (6,8)
Estado civil	
Soltero o unión libre	17 (23,3)
Casado	45 (61,6)
Separado, divorciado o viudo	11 (15,1)
Ocupación	
Empleado	31 (42,5)
Desempleado	26 (35,6)
Pensionado	14 (19,2)
Sin dato	2 (2,7)
Aseguramiento al régimen de salud	
Subsidiado	10 (13,7)
Contributivo	61 (83,6)
Especial	2 (2,7)

La cirrosis criptogénica (19%) y el carcinoma hepatocelular (14%) fueron las indicaciones más frecuentes para el trasplante hepático (tabla 2). Como antecedentes patológicos previos al trasplante se encontró en 32% de los pacientes hipertensión arterial, diabetes en 23%, dislipidemia en 14% y enfermedad cardiovascular en 6%; además 36% de los pacientes consumían alcohol y 25% fumaban cigarrillo.

**Tabla 2:** Características clínicas de los pacientes trasplantados hepáticos del Hospital San Vicente Fundación, Rionegro, Antioquia, Colombia, 2013-2017.

Variable	n (%)
Período del trasplante	
2013	7 (9,6)
2014	15 (20,5)
2015	24 (32,9)
2016	20 (27,4)
2017-I	7 (9,6)
Indicación para el trasplante^a^	
Enfermedad grasa por alcohol	17 (17,9)
Esteatohepatitis no alcohólica	10 (10,5)
Infección por hepatitis C	3 (3,2)
Infección por hepatitis B	3 (3,2)
Hepatitis autoinmune	5 (5,3)
Colangitis biliar primaria	13 (13,7)
Carcinoma hepatocelular	18 (18,9)
Cirrosis criptogénica	19 (20,0)
Otra	7 (7,3)
Terapia inmunosupresora^a^	
Prednisona	33 (18,8)
Tracolimus	45 (25,6)
Ciclosporina	21 (11,9)
Micofenolato	50 (28,4)
Azatioprina	9 (5,1)
Everolimus	18 (10,2)
Causa de muerte	
Choque hipovolémico	1 (16,6)
Choque séptico	4 (66,7)
Insuficiencia renal	1 (16,7)

a Las categorías no son excluyentes

El año con mayor número de trasplantes fue el 2015, con 16%. Los medicamentos más utilizados en las terapias inmunosupresoras fueron micofenolato (50 pacientes), tacrolimus (45 pacientes) y prednisona (33 pacientes), pero hay que tener en cuenta que cada paciente tenía varios medicamentos dentro de su esquema por lo que no se pudo explorar la asociación entre el tratamiento inmunosupresor y el SM. Durante el período de seguimiento de este estudio se reportaron 6 muertes (mortalidad de 8%) y la causa más frecuente de muerte fue choque séptico (67%) (tabla 2).

En la tabla 3 se describe el análisis bivariado que se realizó; solo se encontró asociación entre la aparición de SM con el antecedente de hipertensión arterial (p=0,005) y de diabetes (p=0,013).

**Tabla 3:** Factores asociados a la aparición de síndrome metabólico después del trasplante hepático en pacientes atendidos en el Hospital San Vicente Fundación, Rionegro, Antioquia, Colombia, 2013-2017.

Factor de exposición (antecedente)	Síndrome metabólico		Valor de p
	Sí	No	
Hipertensión arterial			0,005 ^a^
Sí	20	3	
No	28	22	
Diabetes mellitus			0,013 ^a^
Sí	15	2	
No	33	23	
Dislipidemia			0,260 ^b^
Sí	8	2	
No	40	23	
Enfermedad cardiovascular			0,377 ^b^
Sí	3	1	
No	45	24	
Alcohol			0,163 ^a^
Sí	19	7	
No	29	18	
Cigarrillo			0,214 ^a^
Sí	13	5	
No	29	18	

a Prueba de Chi cuadrado

b Prueba exacta de Fisher

## DISCUSIÓN

Dos terceras partes de los pacientes con trasplante hepático atendidos en Hospital de San Vicente Fundación de Rionegro, Antioquia, Colombia, entre 2013 y 2017, desarrollaron SM después del trasplante. Con este trabajo se confirma una vez más que los pacientes trasplantados hepáticos desarrollan con mucha frecuencia SM; sin embargo, la prevalencia de SM encontrada en el presente trabajo (66%) fue casi el doble que la reportada por Thoefner y colaboradores en la revisión sistemática y metanálisis que realizaron en 2015 con 16 publicaciones y 3 539 pacientes con trasplante hepático, en quienes encontraron una prevalencia de SM de 39% (rango 16-64%) y una incidencia de SM de 35% (rango 21-49%) ^10^. Lo que sugiere que los pacientes atendidos en el Centro Especializado San Vicente Fundación de Rionegro, Antioquia, Colombia, pueden presentar alguna condición adicional, que debería ser estudiada, que los hace más propensos a desarrollar SM comparado con los trasplantados hepáticos de otras regiones del mundo.

En este trabajo se encontró que el antecedente de hipertensión arterial fue un factor asociado para la aparición de SM después del trasplante hepático, lo cual coincide con lo reportado por Sprinzl y colaboradores ^11^ con pacientes europeos y Couto y colaboradores ^12^ con pacientes hispánicos y no hispánicos; pero contrasta con otros trabajos que no encontraron dicha asociación con pacientes de diferentes regiones del mundo ^13^ ^-^ ^16^.

El otro factor asociado para el desarrollo de SM postrasplante hepático encontrado en este trabajo fue el antecedente de diabetes, lo cual concuerda con lo reportado previamente en varias investigaciones alrededor del mundo ^11^ ^-^ ^19^ y además con el metanálisis de Thoefner y colaboradores ^10^ se confirma el alto impacto que tiene el antecedente de diabetes en la aparición de SM después del trasplante de hígado (OR = 4,03; IC95% = 2,81-5,80; I^2^ = 0%). 

En este trabajo no fue posible evaluar la asociación entre la terapia inmunosupresora con la incidencia de SM debido a que la terapia incluye la combinación de varios medicamentos y además los pacientes estaban sometidos a cambios tanto de dosis como de medicamentos dentro de la terapia. Sin embargo, la revisión sistemática de Thoefner y colaboradores ^10^ evidenció que los medicamentos inmunosupresores no constituyen un factor de riesgo para el SM después del trasplante hepático.

Se reconocen dos limitaciones principales de este trabajo: 1) el limitado número de historias clínicas que se pudieron incluir en el análisis, 73 de 112, porque contaban con la información completa. 2) No fue posible incluir en el análisis la variable obesidad como posible factor asociado al desarrollo de SM porque en las historias clínicas no se encontró información sobre el índice de masa corporal ni del perímetro abdominal de los pacientes.

En conclusión, en el Centro Especializado San Vicente Fundación de Rionegro, Antioquia, Colombia, se presenta una alta prevalencia de SM postrasplante hepático, incluso mayor que la prevalencia reportada en otras regiones del mundo, y los antecedentes de hipertensión arterial y diabetes constituyen los factores asociados más relevantes para desarrollar dicho síndrome.
